# Supplementary material for: Single-cell division tracing and transcriptomics reveal cell types and differentiation paths in the regenerating lung
Source: Nat Commun. 2024 Mar 12;15:2246. doi: 10.1038/s41467-024-46469-4 (PMC10933319; doi:10.1038/s41467-024-46469-4)
Supplement: Supplementary file 1 — Supplementary Information [file 41467_2024_46469_MOESM1_ESM.pdf]

Single-cell division tracing and transcriptomics reveal cell types and differentiation paths in the regenerating lung

Leila R. Martins, Lina Sieverling, Michelle Michelhans, Chiara Schiller, Cihan Erkut, Thomas G. P. Grünewald, Sergio Triana, Stefan Fröhling, Lars Velten, Hanno Glimm, Claudia Scholl

SUPPLEMENTARY INFORMATION

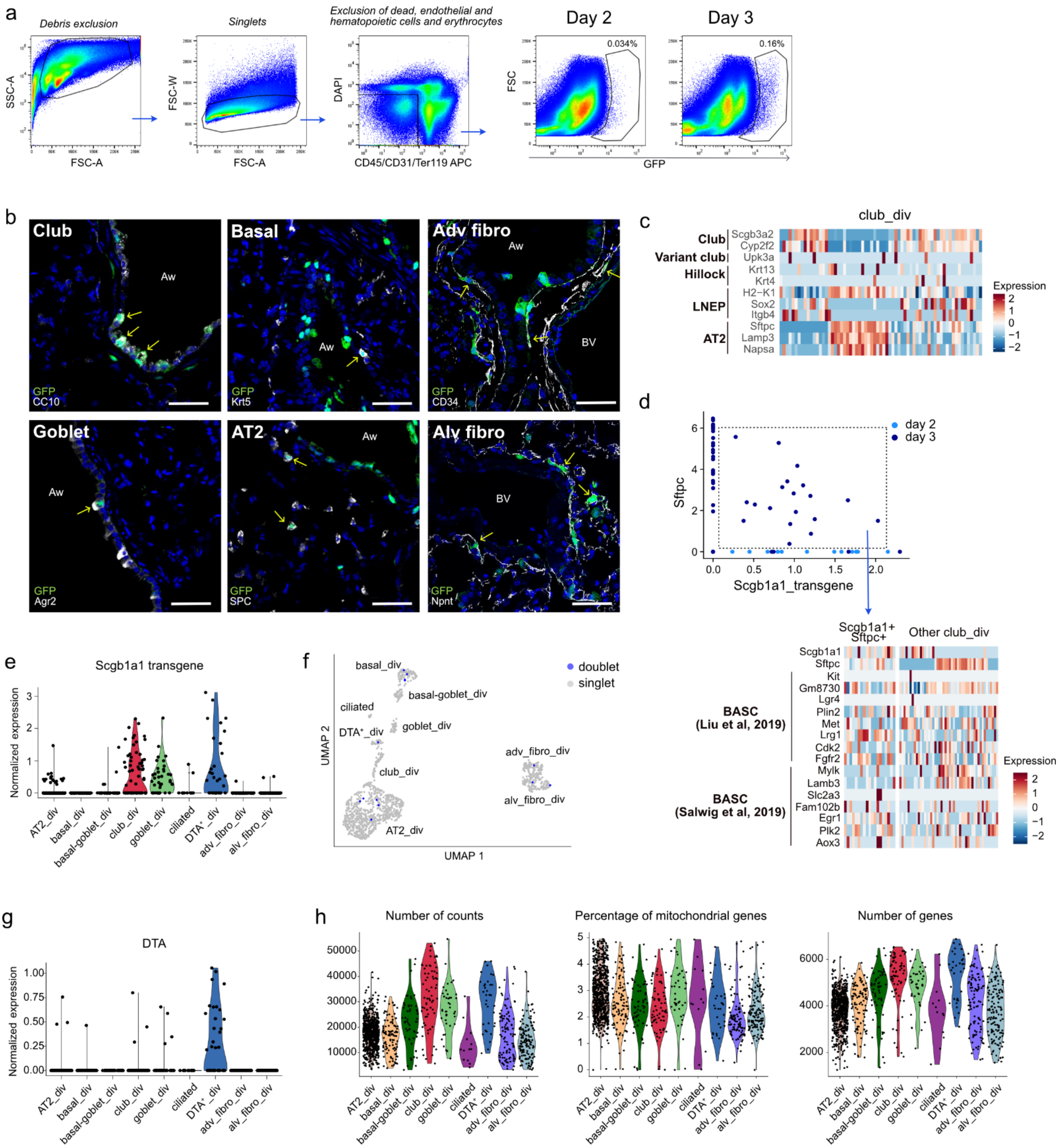

Supplementary Figure 1. Characterization of dividing cells after targeted depletion of *Scgb1a1*⁺ cells.

a. Gating strategy used to sort viable GFP$^+$ cells. **b.** IF staining of dividing GFP$^+$ cells (green) combined with markers for club cells (CC10, white), goblet cells (Agr2, white), basal cells (Krt5, white), AT2 cells (SPC, white), adventitial fibroblasts (adv fibro, CD34, white), and alveolar fibroblasts (alv fibro, Npnt, white). Nuclei are stained with DAPI (blue). All images were taken at day 3 after tamoxifen injection. Aw: airway; BV: blood vessel. Arrows are pointing to GFP$^+$ cells co-stained with cell type specific markers. Representative images of more than three independent experiments with similar results are shown. Scale bar: 50 µm. **c.** Heatmap of the main marker genes for previously described progenitor club cell types, club cells, and AT2 cells in club_div cells at day 2 and 3. **d.** Upper panel: Co-expression of the Scgb1a1 transgene and *Sftpc* in club_div cells. Lower panel: Expression of bronchioalveolar stem cell (BASC) marker genes in club_div cells co-expressing the Scgb1a1 transgene and *Sftpc* and in other club_div cells. **e.** Violin plot showing the expression of the Scgb1a1 transgene in the indicated cell types. **f.** UMAP embedding showing doublet and singlet classification by scDoublet. **g.** Violin plot showing the expression of DTA in the dividing populations at day 2 and 3. **h.** Violin plots showing number of counts, percentage of mitochondrial genes, and number of genes per cell in the different dividing cell populations. The labeling of the y-axis is given in the heading.

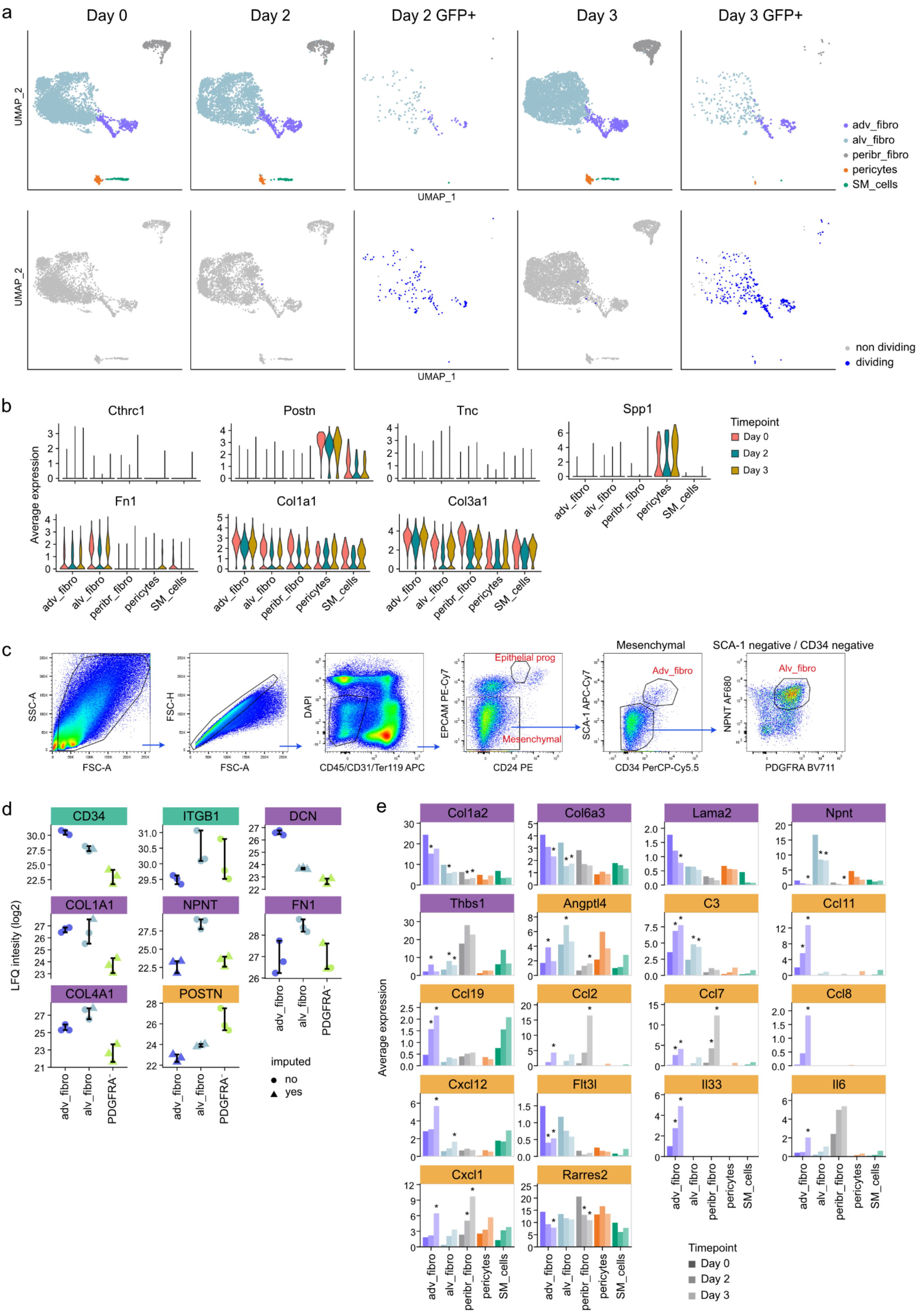
a
Day 0
Day 2
Day 2 GFP+
Day 3
Day 3 GFP+
UMAP_2
UMAP_1
adv_fibro
alv_fibro
peribr_fibro
pericytes
SM_cells
non dividing
dividing
b
Cthrc1
Postn
Tnc
Spp1
Fn1
Col1a1
Col3a1
Average expression
Timepoint
Day 0
Day 2
Day 3
c
SSC-A
FSC-A
FSC-H
DAPI
CD45/CD31/Ter119 APC
EPCAM PE-Cy7
Epithelial prog
Mesenchymal
CD24 PE
SCA-1 APC-Cy7
Adv_fibro
CD34 PerCP-Cy5.5
SCA-1 negative / CD34 negative
NPNT AF680
Alv_fibro
PDGFRA BV711
d
CD34
ITGB1
DCN
COL1A1
NPNT
FN1
COL4A1
POSTN
LFQ intesity (log2)
imputed
no
yes
adv_fibro
alv_fibro
PDGFRA⁻
e
Col1a2
Col6a3
Lama2
Npnt
Thbs1
Angptl4
C3
Ccl11
Ccl19
Ccl2
Ccl7
Ccl8
Cxcl12
Flt3l
Il33
Il6
Cxcl1
Rarres2
Average expression
Timepoint
Day 0
Day 2
Day 3

Supplementary Figure 2. Characterization of mesenchymal cell activation after epithelial injury in SRC mice. **a.** UMAP embedding of mesenchymal cells and dividing GFP^+ mesenchymal cells on the indicated days. **b.** Violin plots showing the expression of fibrosis-associated genes in individual cells of the different mesenchymal populations on day 0, 2, and 3. **c.** Gating strategy used to sort epithelial progenitor cells (epithelial prog), adventitial fibroblasts (adv_fibro), and alveolar fibroblasts (alv_fibro). **d.** Protein expression in log2 label-free quantification (LFQ) intensity of upregulated ligands in adventitial fibroblasts, alveolar fibroblasts, and PDGFRA negative cells (see Fig. 2f) at day 0 for which protein data were available. Three independent experiments are shown. Error bars denote standard deviation. **e.** Average normalized RNA expression of all ligands in the modified CellChat ligand-receptor database that are differentially expressed between day 0 and day 2 or day 3 in adv_fibro by scRNA-seq. *Bmp4*, *Tgfb3,* and *Mif* (included in Fig. 2g) were excluded from this figure. Asterisks denote significant differential expression on day 2 or 3 compared to day 0 (MAST test, FC >=1.5, p_adj <=0.05). **d, e.** Ligand genes are sorted by the possible type of interaction (yellow: secreted signaling, purple: ECM receptor, green: cell-cell contact). **a, b, e.** scRNA-seq analysis was done using total mesenchymal cells on day 0 (n=2 mice), day 2 (n=3 mice), and day 3 (n=3 mice), and GFP^+ mesenchymal cells on day 2 (n=12 mice) and day 3 (n=3 mice).

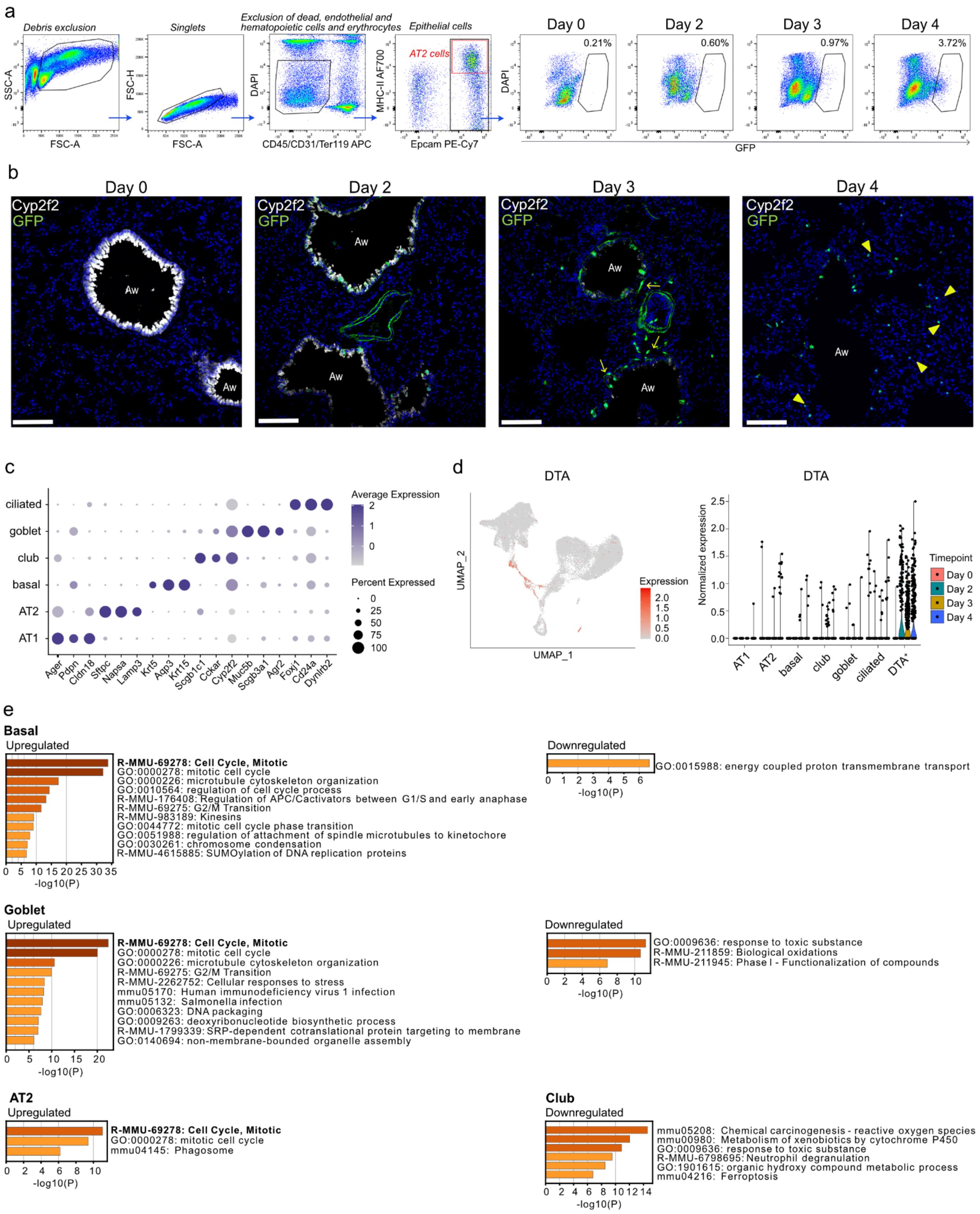
a
Debris exclusion
Singlets
Exclusion of dead, endothelial and hematopoietic cells and erythrocytes
Epithelial cells
AT2 cells
Day 0
Day 2
Day 3
Day 4
0.21%
0.60%
0.97%
3.72%
SSC-A
FSC-A
FSC-H
DAPI
CD45/CD31/Ter119 APC
MHC-II AF700
Epcam PE-Cy7
GFP
b
Cyp2f2
GFP
Aw
c
ciliated
goblet
club
basal
AT2
AT1
Ager Pdpn Cldn18 Sftpc Napsa Lamp3 Krt5 Aqp3 Krt15 Scgb1c1 Cckar Cyp2f2 Muc5b Scgb3a1 Agr2 Foxj1 Cd24a Dynlrb2
Average Expression
Percent Expressed
0
25
50
75
100
d
DTA
UMAP_1
UMAP_2
Expression
2.0
1.5
1.0
0.5
0.0
Normalized expression
2.5
AT1 AT2 basal club goblet ciliated DTA+
Timepoint
Day 0
Day 2
Day 3
Day 4
e
Basal
Upregulated
R-MMU-69278: Cell Cycle, Mitotic
GO:0000278: mitotic cell cycle
GO:0000226: microtubule cytoskeleton organization
GO:0010564: regulation of cell cycle process
R-MMU-176408: Regulation of APC/Cactivators between G1/S and early anaphase
R-MMU-69275: G2/M Transition
R-MMU-983189: Kinesins
GO:0044772: mitotic cell cycle phase transition
GO:0051988: regulation of attachment of spindle microtubules to kinetochore
GO:0030261: chromosome condensation
R-MMU-4615885: SUMOylation of DNA replication proteins
0 5 10 15 20 25 30 35
-log10(P)
Downregulated
GO:0015988: energy coupled proton transmembrane transport
0 1 2 3 4 5 6
-log10(P)
Goblet
Upregulated
R-MMU-69278: Cell Cycle, Mitotic
GO:0000278: mitotic cell cycle
GO:0000226: microtubule cytoskeleton organization
R-MMU-69275: G2/M Transition
R-MMU-2262752: Cellular responses to stress
mmu05170: Human immunodeficiency virus 1 infection
mmu05132: Salmonella infection
GO:0006323: DNA packaging
GO:0009263: deoxyribonucleotide biosynthetic process
R-MMU-1799339: SRP-dependent cotranslational protein targeting to membrane
GO:0140694: non-membrane-bounded organelle assembly
0 5 10 15 20
-log10(P)
Downregulated
GO:0009636: response to toxic substance
R-MMU-211859: Biological oxidations
R-MMU-211945: Phase I - Functionalization of compounds
0 2 4 6 8 10
-log10(P)
AT2
Upregulated
R-MMU-69278: Cell Cycle, Mitotic
GO:0000278: mitotic cell cycle
mmu04145: Phagosome
0 2 4 6 8 10
-log10(P)
Club
Downregulated
mmu05208: Chemical carcinogenesis - reactive oxygen species
mmu00980: Metabolism of xenobiotics by cytochrome P450
GO:0009636: response to toxic substance
R-MMU-6798695: Neutrophil degranulation
GO:1901615: organic hydroxy compound metabolic process
mmu04216: Ferroptosis
0 2 4 6 8 10 12 14
-log10(P)

f

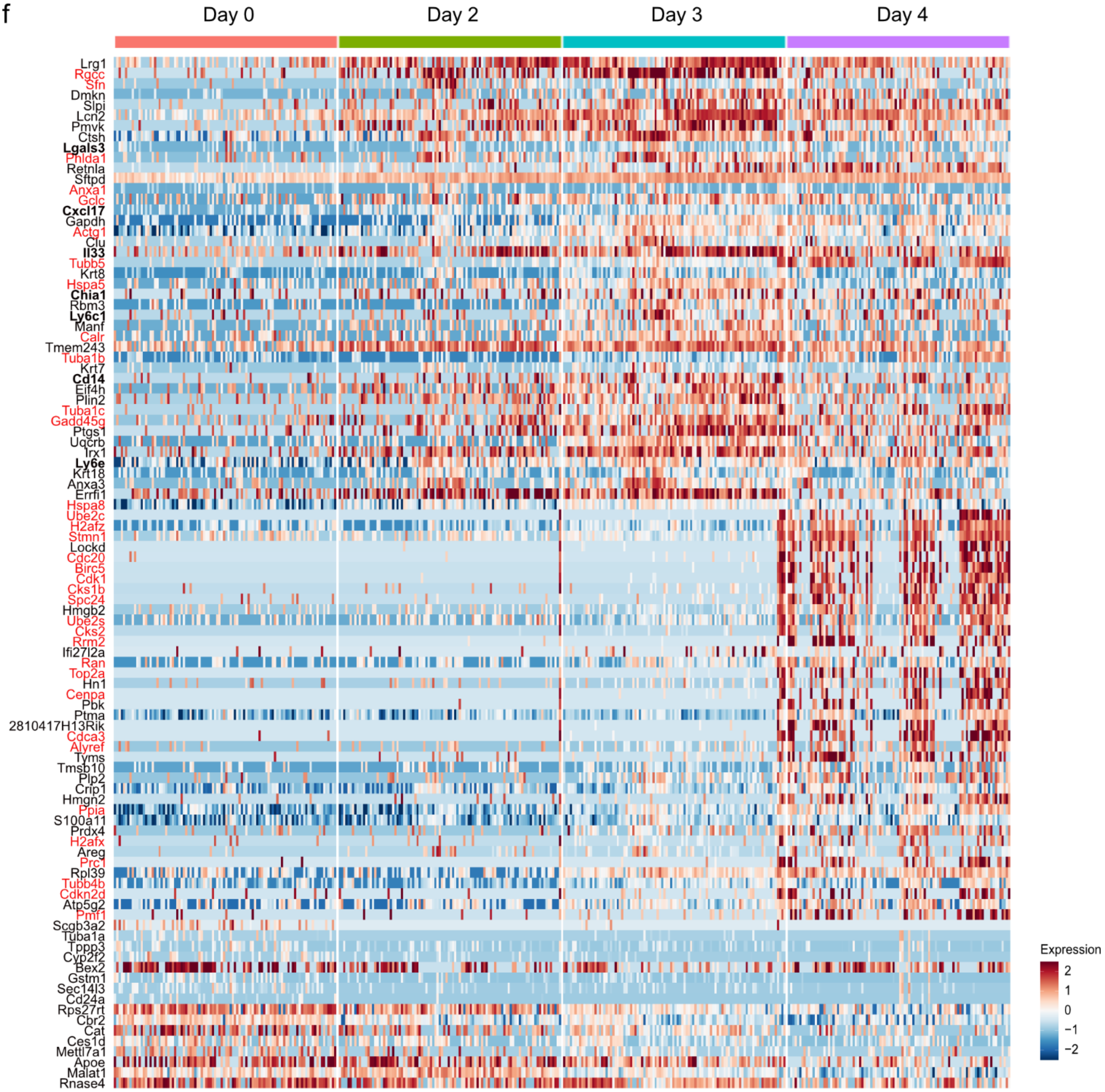

g

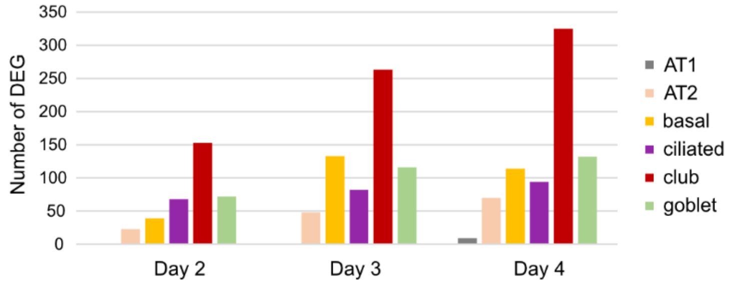

Supplementary Figure 3. Transcriptional changes in epithelial lung cells after *Scgb1a1*⁺ cell depletion.
a. Gating strategy used to sort epithelial cells, while keeping only 10% of AT2 cells (MHC II$^+$) in relation to the total number of cells (red gate), and gating strategy to sort GFP$^+$ epithelial cells. Percentage of GFP$^+$ epithelial cells in relation to total epithelial cells on day 0 to 4 is indicated. **b.** IF staining of SRC mouse lungs with Cyp2f2 (white; club cells), CycB1-GFP (green; dividing cells), and DAPI (blue; nuclei) before (day 0) and 2, 3, and 4 days after injecting tamoxifen on two consecutive days. GFP$^+$ spindle-like cells (arrows) and GFP$^+$ alveolar cells (arrow heads) can be observed from day 3. Aw: airway. Scale bar: 100 µm. Images are representative of at least three independent experiments with n=3 animals per timepoint. **c.** Dot plot showing the average scaled expression and the percentage of cells expressing cell type-specific marker genes across the epithelial populations. **d.** UMAP embedding (left) and violin plot (right) showing

the expression of DTA in epithelial cell types from day 0 to day 4. **e.** GSEA of DEGs on day 2, 3, and 4 compared to day 0 (MAST test, FC >=2, p_adj <0.05) in the different epithelial populations. **f.** Expression of DEGs in AT2 cells through time (MAST test, FC >2, p_adj <0.05). Genes in bold are mentioned in the text. Genes in red are related to cell division. **g.** Number of DEGs at the indicated days as compared to day 0 for all epithelial cell types (MAST test, FC >2, p_adj <0.05).

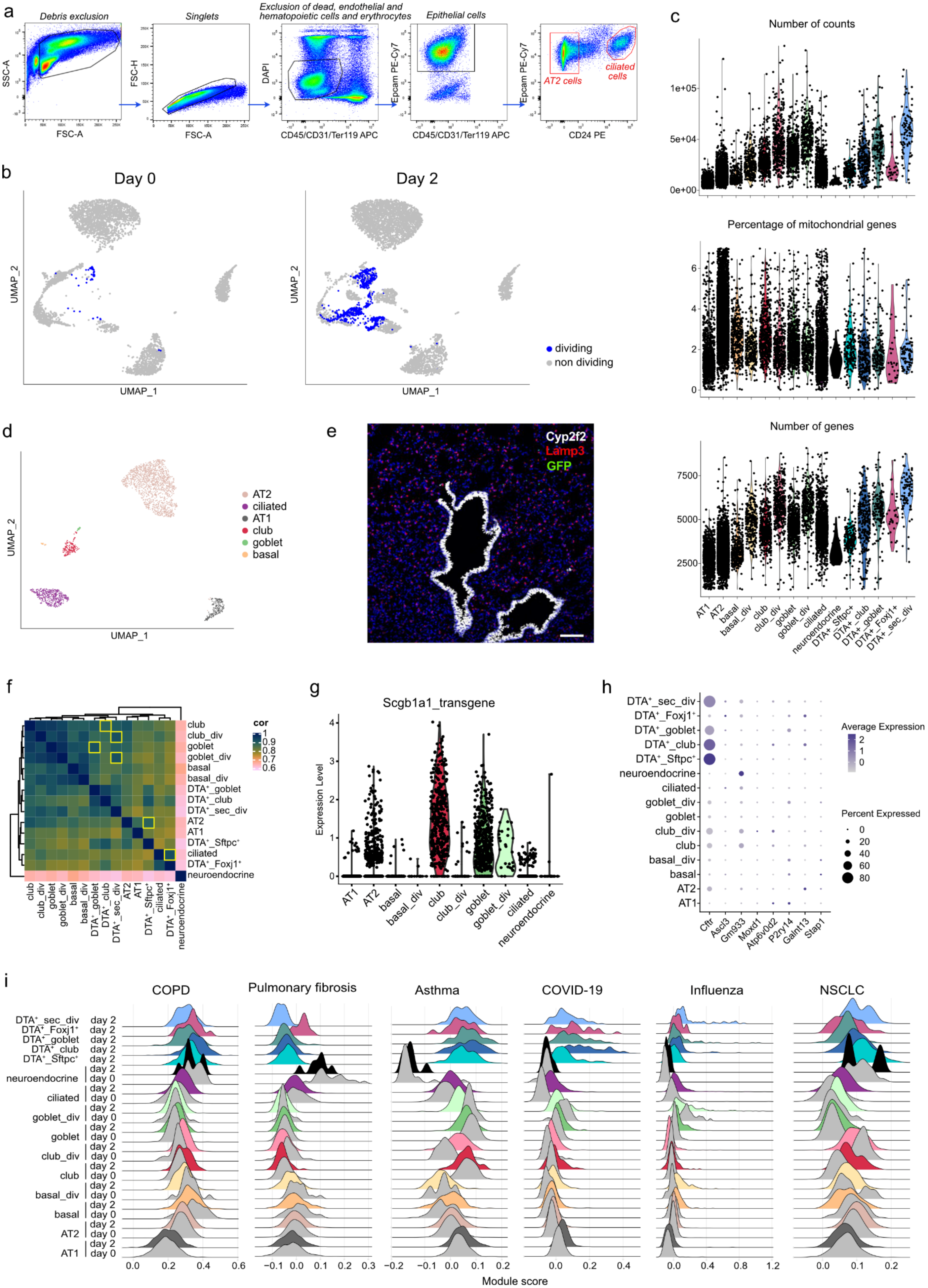
a
Debris exclusion
Singlets
Exclusion of dead, endothelial and hematopoietic cells and erythrocytes
Epithelial cells
SSC-A
FSC-A
FSC-H
DAPI
CD45/CD31/Ter119 APC
Epcam PE-Cy7
CD24 PE
AT2 cells
ciliated cells
b
Day 0
Day 2
UMAP_2
UMAP_1
dividing
non dividing
c
Number of counts
Percentage of mitochondrial genes
Number of genes
AT1
AT2
basal
basal_div
club
club_div
goblet
goblet_div
ciliated
neuroendocrine
DTA+ Sftpc+
DTA+_club
DTA+_goblet
DTA+_Foxj1+
DTA+_sec_div
d
AT2
ciliated
AT1
club
goblet
basal
e
Cyp2f2
Lamp3
GFP
f
cor
g
Scgb1a1_transgene
Expression Level
h
Average Expression
Percent Expressed
Cftr
Ascl3
Gm933
Moxd1
Atp6v0d2
P2ry14
Galnt13
Stap1
i
COPD
Pulmonary fibrosis
Asthma
COVID-19
Influenza
NSCLC
day 2
day 0
Module score

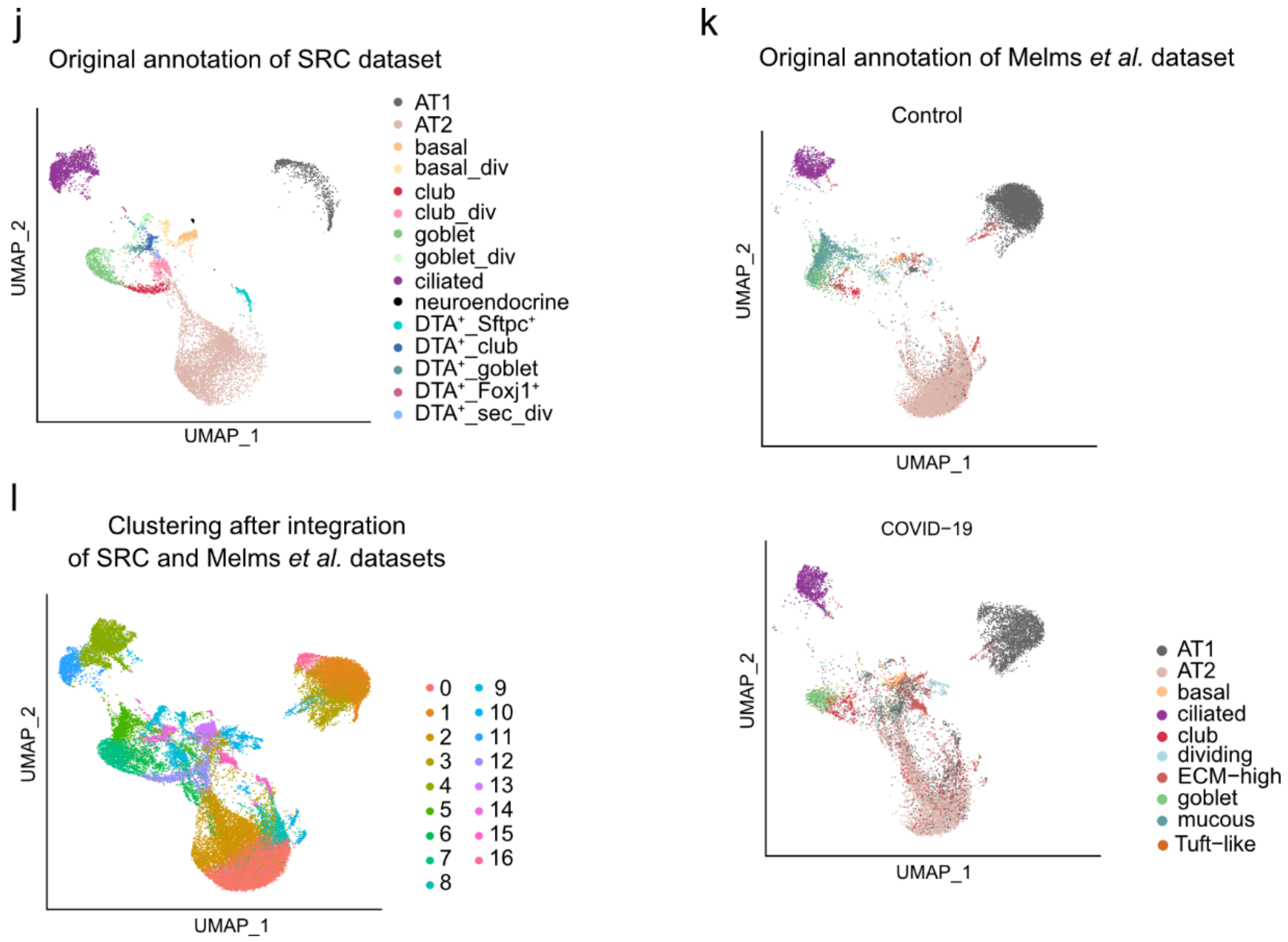

Supplementary Figure 4. Characterization of DTA$^+$ epithelial cells and comparison to transcriptional profiles of human lung diseases.

a. Gating strategy used to sort epithelial cells, while keeping only 10% of AT2 cells (CD24$^-$) and 10% of ciliated cells (CD24^{high}) in relation to the total number of cells (red gates). **b.** UMAP embedding showing the distribution of dividing and non-dividing cells. **c.** Violin plots showing number of counts, percentage of mitochondrial genes, and number of genes in each cell types**.** The labeling of the y-axis is given in the heading. **d.** UMAP embedding showing epithelial cell types in Rosa26R-DTA x CycB1-GFP control mice (n=9) on day 2 after two consecutive tamoxifen injections. The sorting and sequencing strategy was the same as for SRC mice. **e.** IF staining of Rosa26R-DTA x CycB1-GFP control mouse lungs with Cyp2f2 (white; club cells), Lamp3 (red; AT2 cells), CycB1-GFP (green; dividing cells), and DAPI (blue; nuclei) on day 2 after injecting tamoxifen. Scale bar: 100 µm. Images are representative of n=3 mice. **f.** Heatmap of the transcriptome Spearman correlation coefficient (cor) between all epithelial cell types on day 2. Yellow boxes highlight the closest population to each DTA$^+$ cell type. **g.** Violin plot of the Scgb1a1 transgene expression on day 0 across all epithelial cells in SRC mice. **h.** Dot plot of ionocyte marker gene expression in all epithelial cell populations at day 2. **i.** Ridge plots showing the expression of human lung disease signatures in SRC mouse epithelial cells on day 0 and day 2. Gene signatures for asthma, non-small cell lung carcinoma, and influenza were taken from DisGeNET v7 [1], and chronic obstructive pulmonary disease [2], COVID-19 bronchial epithelial cells [3], and pulmonary fibrosis [4] from MSigDB v7.5.1 [5, 6]. **j-l.** UMAP embedding after integration of SRC and Melms *et al.* [7] datasets showing the original annotation of cells from the SRC dataset (**j**), the original annotation of cells from the Melms et al. dataset (**k**), and re-clustering of both datasets (**l**).

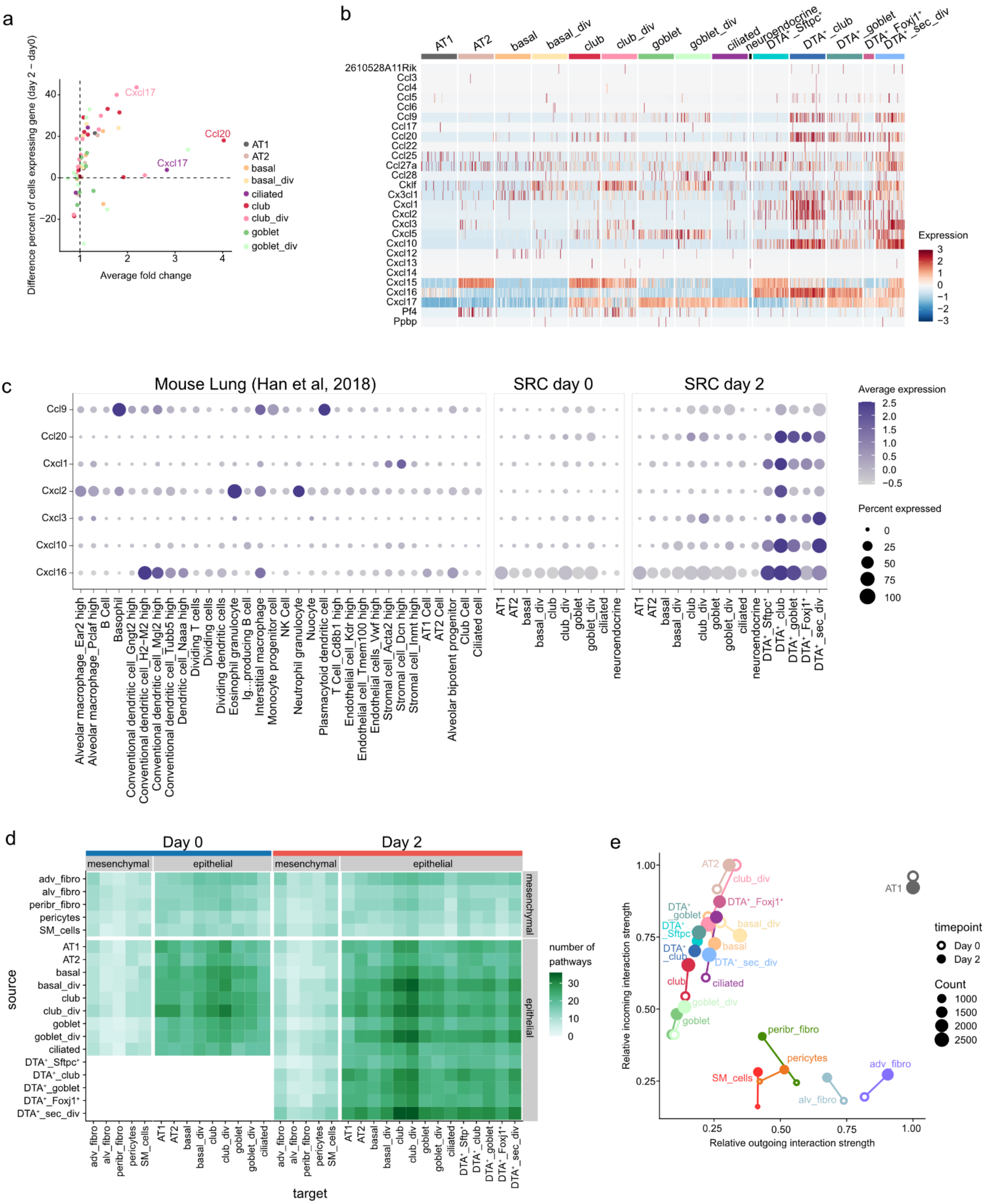
a
b
c
d
e
Difference percent of cells expressing gene (day 2 – day0)
Average fold change
Cxcl17
Ccl20
Cxcl17
Expression
Mouse Lung (Han et al, 2018)
SRC day 0
SRC day 2
Average expression
Percent expressed
Day 0
Day 2
mesenchymal
epithelial
number of pathways
source
target
Relative incoming interaction strength
Relative outgoing interaction strength
timepoint
Count

f

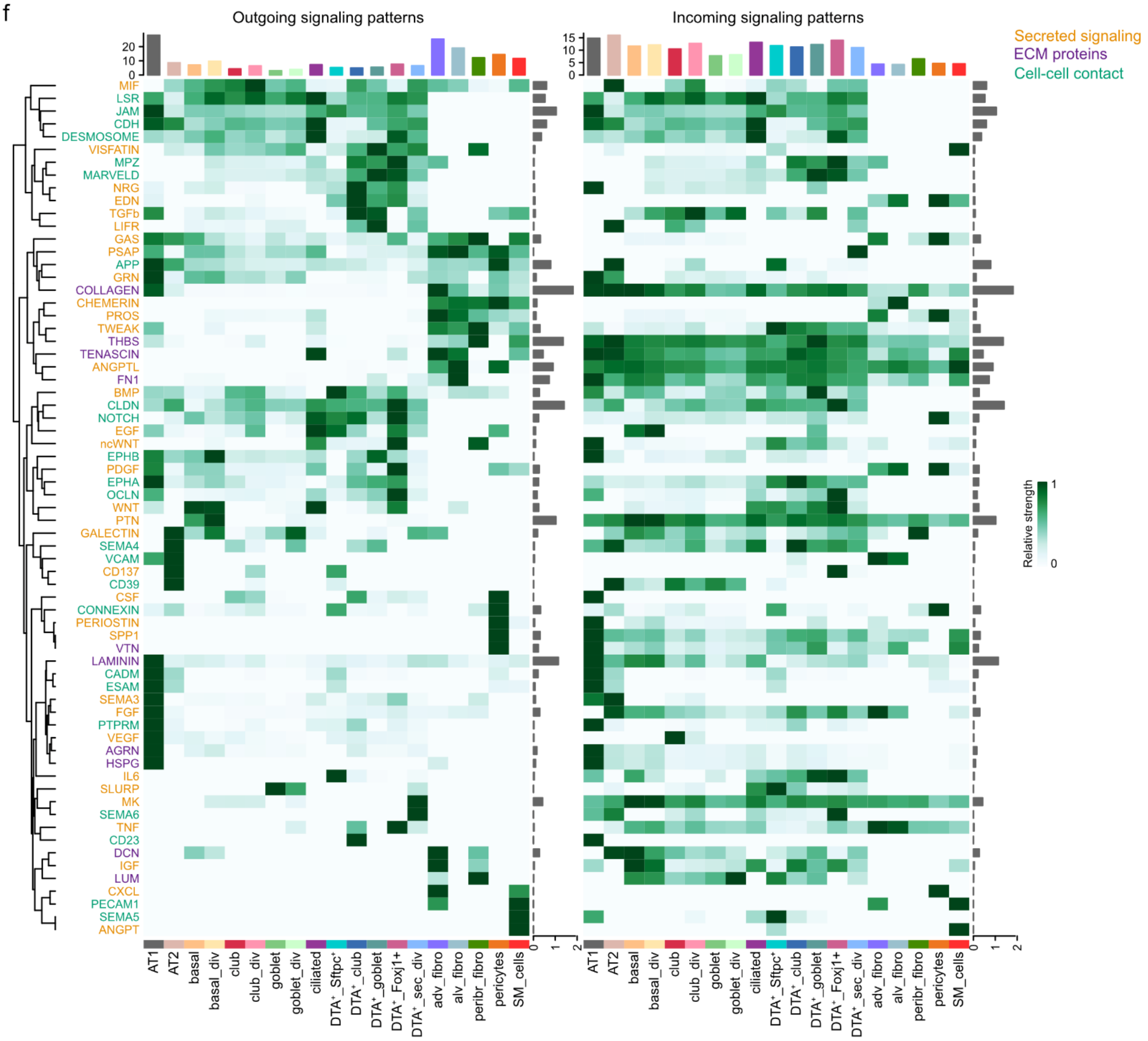

Outgoing signaling patterns
Incoming signaling patterns
Secreted signaling
ECM proteins
Cell-cell contact
MIF
LSR
JAM
CDH
DESMOSOME
VISFATIN
MPZ
MARVELD
NRG
EDN
TGFb
LIFR
GAS
PSAP
APP
GRN
COLLAGEN
CHEMERIN
PROS
TWEAK
THBS
TENASCIN
ANGPTL
FN1
BMP
CLDN
NOTCH
EGF
ncWNT
EPHB
PDGF
EPHA
OCLN
WNT
PTN
GALECTIN
SEMA4
VCAM
CD137
CD39
CSF
CONNEXIN
PERIOSTIN
SPP1
VTN
LAMININ
CADM
ESAM
SEMA3
FGF
PTPRM
VEGF
AGRN
HSPG
IL6
SLURP
MK
SEMA6
TNF
CD23
DCN
IGF
LUM
CXCL
PECAM1
SEMA5
ANGPT
Relative strength
AT1
AT2
basal
basal_div
club
club_div
goblet
goblet_div
ciliated
DTA+_Sftpc+
DTA+_club
DTA+_goblet
DTA+_Foxj1+
DTA+_sec_div
adv_fibro
alv_fibro
peribr_fibro
pericytes
SM_cells

g

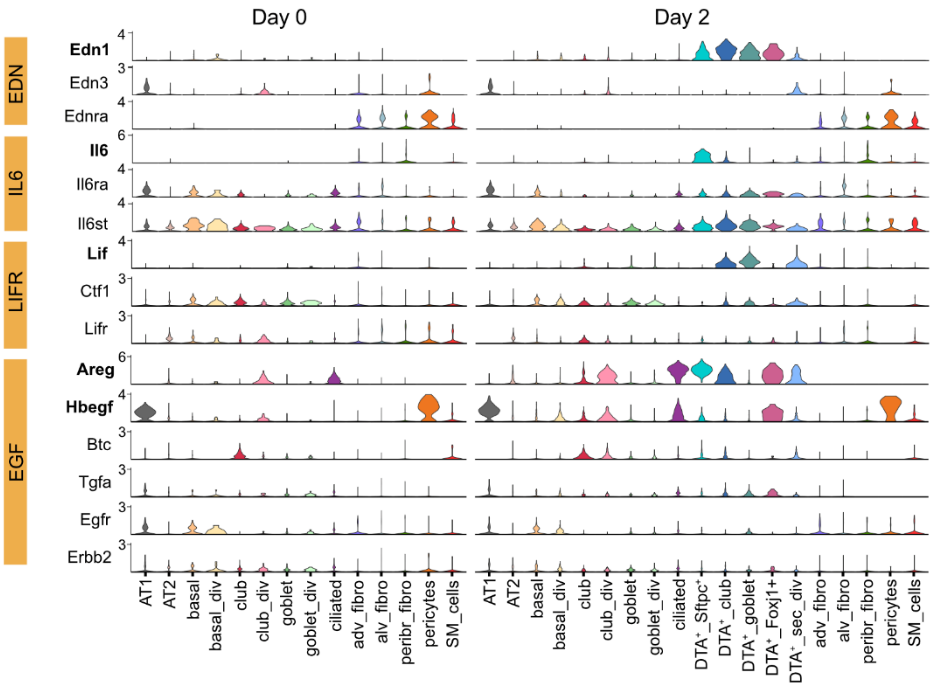

Day 0
Day 2
EDN
IL6
LIFR
EGF
Edn1
Edn3
Ednra
Il6
Il6ra
Il6st
Lif
Ctf1
Lifr
Areg
Hbegf
Btc
Tgfa
Egfr
Erbb2
AT1
AT2
basal
basal_div
club
club_div
goblet
goblet_div
ciliated
adv_fibro
alv_fibro
peribr_fibro
pericytes
SM_cells
DTA+_Sftpc+
DTA+_club
DTA+_goblet
DTA+_Foxj1+
DTA+_sec_div

Supplementary Figure 5. Chemokine expression and cellular crosstalk in the SRC mouse model.
a. Differential expression of chemokines in epithelial lung cells upon injury. Only genes detected in at least 20% of cells in a population in either of the timepoints are shown. Genes with FC > 2 and p_adj < 0.05 are labeled (MAST test). **b.** Heatmap of chemokine expression in epithelial lung cells on day 2. Scaling of expression was done after downsampling to 100 cells per cell type. Chemokines without counts in any of the cell types were excluded. **c.** Dot plot showing the average scaled expression and the percentage of cells expressing selected chemokines (see Fig. 5b) in lung cells in homeostasis from the mouse atlas of Han *et al.* [8] and in epithelial lung cells of the SRC model on day 0 and day 2. **d.** Number of signaling pathways between any two cell types before (day 0) and after tamoxifen administration (day 2) of the modified CellChat ligand-receptor database. **e.** Summed incoming and outgoing interaction strength of each cell type. The interaction strength was scaled to the maximum summed interaction strength at the respective timepoint. Circle size is proportional to the number of significant ligand-receptor pairs of the respective cell type at that timepoint. **f.** Heatmaps of outgoing (left) and incoming (right) signaling patterns per cell type on day 2. The signaling strengths are scaled to the maximum of each row. Column annotations show the summed absolute signaling strengths of each cell type. Row annotations show the log information flow (i.e. summed signaling strength) of each pathway. Signaling pathways are colored by the type of interaction (yellow: secreted signaling, purple: ECM proteins, green: cell-cell contact) and sorted according to the dendrogram of outgoing signaling patterns. **g.** Violin plots showing normalized expression of the main ligand and receptor genes contributing to the EDN, IL6, LIFR, and EGF signaling pathways on day 0 and day2.

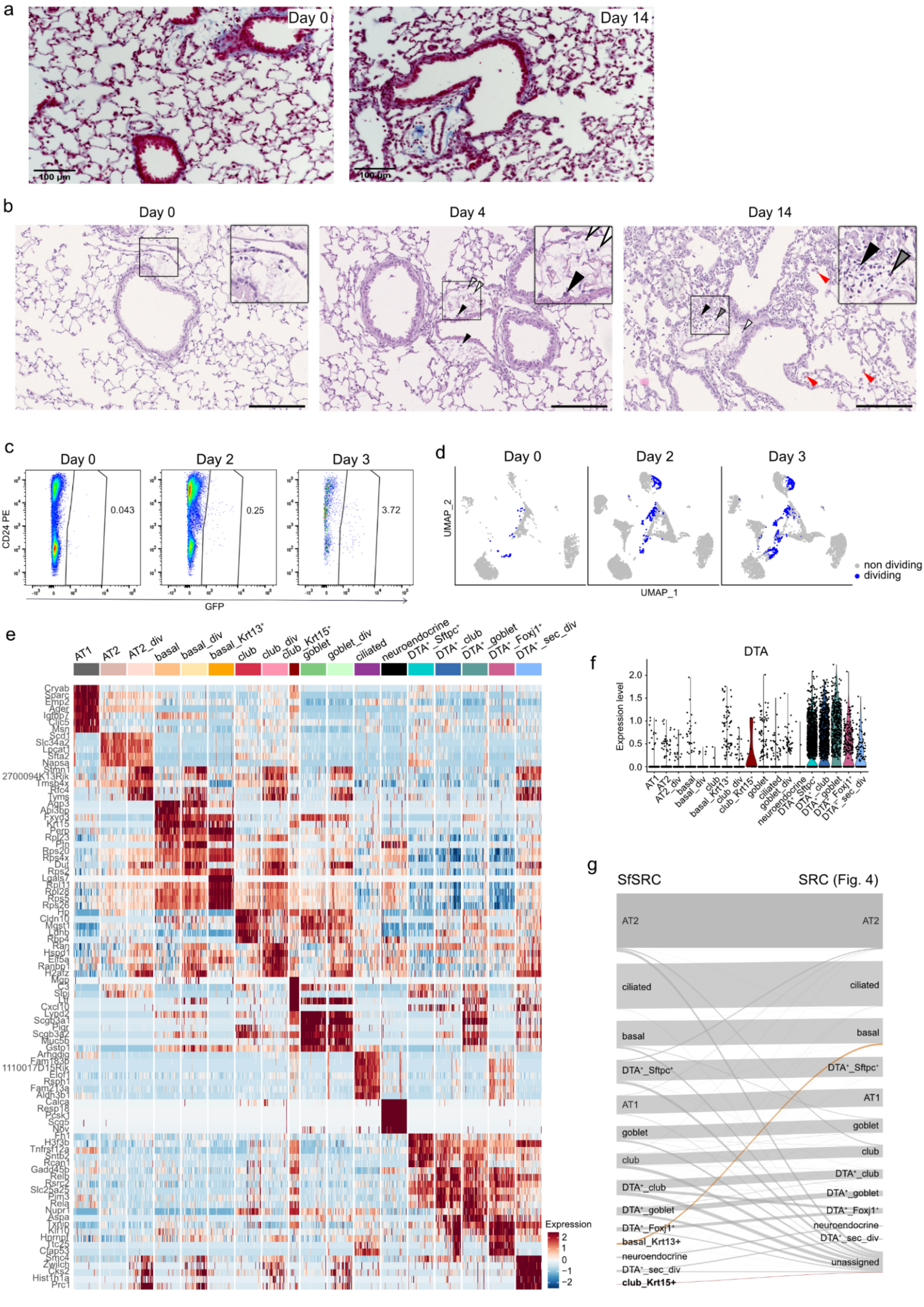

a
Day 0
Day 14
100 µm
100 µm
b
Day 0
Day 4
Day 14
c
Day 0
Day 2
Day 3
CD24 PE
GFP
0.043
0.25
3.72
d
Day 0
Day 2
Day 3
UMAP_2
UMAP_1
non dividing
dividing
e
AT1
AT2
AT2_div
basal
basal_div
basal_Krt13+
club
club_div
club_Krt15+
goblet
goblet_div
ciliated
neuroendocrine
DTA+_Sftpc+
DTA+_club
DTA+_goblet
DTA+_Foxj1+
DTA+_sec_div
Expression
f
DTA
Expression level
g
SfSRC
SRC (Fig. 4)
AT2
ciliated
basal
DTA+_Sftpc+
AT1
goblet
club
DTA+_club
DTA+_goblet
DTA+_Foxj1+
basal_Krt13+
neuroendocrine
DTA+_sec_div
club_Krt15+
unassigned

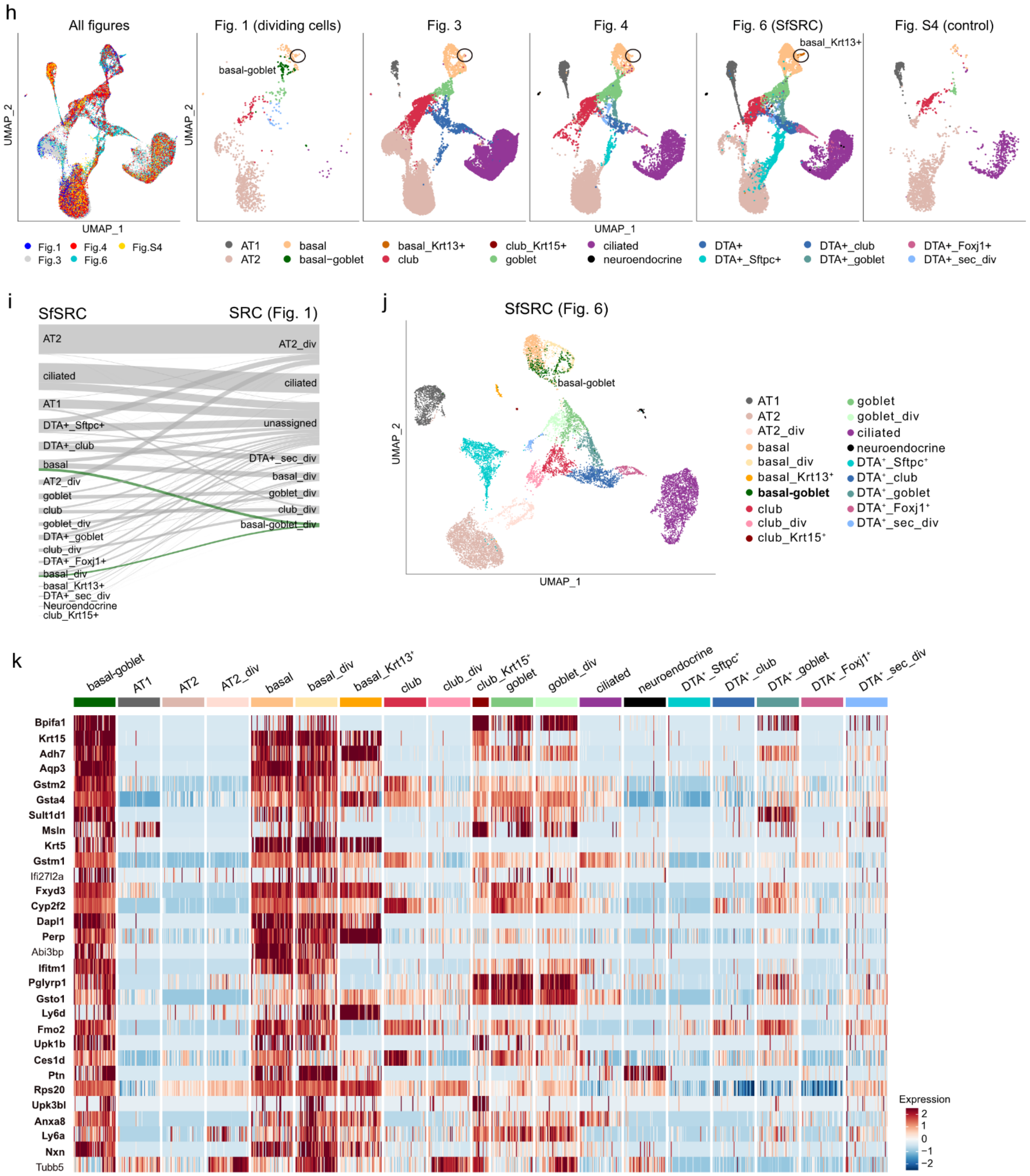

Supplementary Figure 6. Characterization of lung epithelial cells in the SfSRC mouse model.
a. Masson staining of SfSRC mouse lungs before (day 0) and 14 days after two consecutive tamoxifen injections. Images are representative of n=3 mice per timepoint and two independent experiments. Scale bar: 100 µm. **b.** H & E staining of SfSRC mouse lungs before (day 0), and 4 and 14 days after two consecutive tamoxifen injections. Images are representative of one experiment with n=2 mice per timepoint. Black arrowhead: plasma cell; white arrowhead: lymphocyte; gray arrowhead: neutrophil granulocyte; red arrowhead: alveolar macrophage. Scale bar: 200 µm. Insets on the top right on each picture are 2x magnifications of the demarcated area in the main picture. **c.** Flow cytometry plots showing the percentage of GFP$^+$ epithelial cells in relation to total epithelial cells before (day 0) and two and three days after tamoxifen injection in SfSRC mouse lungs. **d.** UMAP embedding showing dividing and non-dividing cells on day 0, 2, and 3. **e.** Heatmap of the top five marker genes ranked by power across all epithelial populations (all three timepoints merged). **f.** Violin plot showing the expression of DTA in the indicated cell types (all three timepoints merged). **g.** Sankey diagram of SfSRC mouse epithelial cells projected onto SRC mouse epithelial cells using scmap. **h.** UMAP

embedding of all scRNA-seq datasets integrated (left), and the same UMAP showing the original cell type annotation in the different figures without discriminating cell cycle status. Circles depict the location of the basal_Krt13^{+} cell population. **i.** Sankey diagram of SfSRC mouse epithelial cells projected onto SRC mouse epithelial dividing cells using scmap. **j.** UMAP embedding of scRNA-seq data presented in Fig. 6 showing the distribution of basal-goblet cells (identified in i) in relation to other cell types. **k.** Heatmap of the top 30 upregulated genes in basal-goblet cells of the SfSRC model (identified in i) ranked by fold change (test= MAST, p_adj <0.05). Genes in bold are common markers to basal-goblet_div cells from Fig.1.

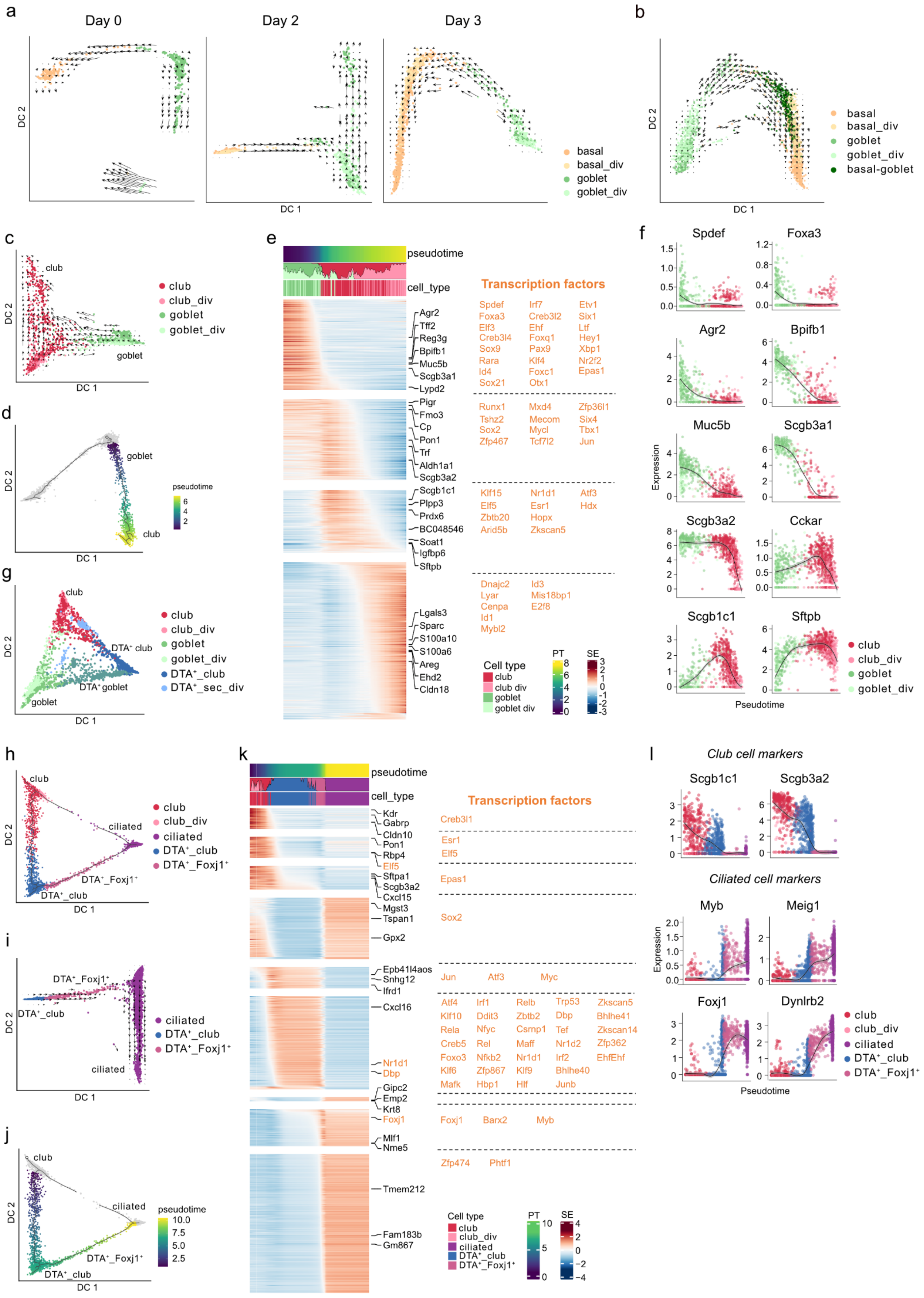

a
Day 0
Day 2
Day 3
DC 2
DC 1
basal
basal_div
goblet
goblet_div
b
basal-goblet
c
club
club_div
d
pseudotime
e
cell_type
Transcription factors
Agr2
Tff2
Reg3g
Bpifb1
Muc5b
Scgb3a1
Lypd2
Pigr
Fmo3
Cp
Pon1
Trf
Aldh1a1
Scgb3a2
Scgb1c1
Plpp3
Prdx6
BC048546
Soat1
Igfbp6
Sftpb
Lgals3
Sparc
S100a10
S100a6
Areg
Ehd2
Cldn18
Spdef Irf7 Etv1
Foxa3 Creb3l2 Six1
Elf3 Ehf Ltf
Creb3l4 Foxq1 Hey1
Sox9 Pax9 Xbp1
Rara Klf4 Nr2f2
Id4 Foxc1 Epas1
Sox21 Otx1
Runx1 Mxd4 Zfp36l1
Tshz2 Mecom Six4
Sox2 Mycl Tbx1
Zfp467 Tcf7l2 Jun
Klf15 Nr1d1 Atf3
Elf5 Esr1 Hdx
Zbtb20 Hopx
Arid5b Zkscan5
Dnajc2 Id3
Lyar Mis18bp1
Cenpa E2f8
Id1
Mybl2
Cell type
club div
goblet div
PT
SE
f
Spdef
Foxa3
Agr2
Bpifb1
Muc5b
Scgb3a1
Scgb3a2
Cckar
Scgb1c1
Sftpb
Expression
Pseudotime
g
DTA⁺ club
DTA⁺ goblet
DTA⁺_club
DTA⁺_sec_div
h
ciliated
DTA⁺_Foxj1⁺
i
j
k
Kdr
Gabrp
Cldn10
Pon1
Rbp4
Elf5
Sftpa1
Scgb3a2
Cxcl15
Mgst3
Tspan1
Gpx2
Epb41l4aos
Snhg12
Ifrd1
Cxcl16
Nr1d1
Dbp
Gipc2
Emp2
Krt8
Foxj1
Mlf1
Nme5
Tmem212
Fam183b
Gm867
Creb3l1
Esr1
Elf5
Epas1
Sox2
Jun Atf3 Myc
Atf4 Irf1 Relb Trp53 Zkscan5
Klf10 Ddit3 Zbtb2 Dbp Bhlhe41
Rela Nfyc Csrnp1 Tef Zkscan14
Creb5 Rel Maff Nr1d2 Zfp362
Foxo3 Nfkb2 Nr1d1 Irf2 EhfEhf
Klf6 Zfp867 Klf9 Bhlhe40
Mafk Hbp1 Hlf Junb
Foxj1 Barx2 Myb
Zfp474 Phtf1
l
Club cell markers
Scgb1c1
Scgb3a2
Ciliated cell markers
Myb
Meig1
Foxj1
Dynlrb2

Supplementary Figure 7. Trajectory analysis of lung epithelial cells in the SfSRC mouse model.
a. Diffusion maps of basal and goblet cells with RNA velocity vectors calculated individually for each timepoint. **b.** Diffusion map of basal, goblet, and basal-goblet cells with RNA velocity vectors indicating transition from goblet to basal through basal-goblet cells. **c.** Diffusion map of goblet and club cells with RNA velocity vectors indicating differentiation of goblet into club cells. **d.** Diffusion map of basal, goblet, and club cells with pseudotime trajectory colored by pseudotime of cells selected to characterize the goblet to club cell trajectory in e. **e.** Smoothed expression heatmap of the top 1,000 altered genes along the differentiation trajectory from goblet to club cells. The names of the top seven genes in each cluster are annotated and transcription factors are marked in orange. All transcription factors of each cluster are listed on the right. **f.** Normalized expression along the goblet to club cell pseudotime trajectory for exemplary genes. The black line is the smoothed expression using local polynomial regression fitting, with the 95% confidence interval shown in gray. **g.** Diffusion map of all secretory cell types. **h.** Diffusion map of club, DTA$^+$_club, DTA$^+$_Foxj1$^+$, and ciliated cells with pseudotime trajectory colored by cell type. **i.** Diffusion map of DTA$^+$_club, DTA$^+$_Foxj1$^+$, and ciliated cells with RNA velocity vectors indicating transition of DTA$^+$_club into DTA$^+$_Foxj1$^+$ cells. **j.** Diffusion map of club, DTA$^+$_club, DTA$^+$_Foxj1$^+$, and ciliated cells colored by pseudotime of cells selected to characterize the club to ciliated cell trajectory in k. **k.** Smoothed expression heatmap of the top 1,000 altered genes along the differentiation trajectory from club to ciliated cells. The names of the top three genes in each cluster are annotated and transcription factors are marked in orange. All transcription factors of each cluster are listed on the right. **l.** Normalized expression along the club to ciliated cell pseudotime trajectory for exemplary genes. Smoothed expression and confidence interval are shown as in f. Figures were calculated on merged expression data of the SfSRC model from all timepoints (day 0, 2, and 3). PT: pseudotime; SE: scaled expression.

References

1. Pinero J*, et al.* The DisGeNET knowledge platform for disease genomics: 2019 update. *Nucleic Acids Res* **48**, D845-D855 (2020).

2. Ning W*, et al.* Comprehensive gene expression profiles reveal pathways related to the pathogenesis of chronic obstructive pulmonary disease. *Proc Natl Acad Sci U S A* **101**, 14895-14900 (2004).

3. Blanco-Melo D*, et al.* Imbalanced Host Response to SARS-CoV-2 Drives Development of COVID-19. *Cell* **181**, 1036-1045 e1039 (2020).

4. Kohler S*, et al.* The Human Phenotype Ontology in 2021. *Nucleic Acids Res* **49**, D1207-D1217 (2021).

5. Subramanian A*, et al.* Gene set enrichment analysis: a knowledge-based approach for interpreting genome-wide expression profiles. *Proc Natl Acad Sci U S A* **102**, 15545-15550 (2005).

6. Liberzon A, Birger C, Thorvaldsdottir H, Ghandi M, Mesirov JP, Tamayo P. The Molecular Signatures Database (MSigDB) hallmark gene set collection. *Cell Syst* **1**, 417-425 (2015).

7. Melms JC*, et al.* A molecular single-cell lung atlas of lethal COVID-19. *Nature* **595**, 114-119 (2021).

8. Han X*, et al.* Mapping the Mouse Cell Atlas by Microwell-Seq. *Cell* **172**, 1091-1107 e1017 (2018).
