# Supplementary material for: Single-cell division tracing and transcriptomics reveal cell types and differentiation paths in the regenerating lung
Source: Nat Commun. 2024 Mar 12;15:2246. doi: 10.1038/s41467-024-46469-4 (PMC10933319; doi:10.1038/s41467-024-46469-4)
Supplement: Supplementary file 3 — Description of Additional Supplementary Files [file 41467_2024_46469_MOESM3_ESM.docx]

**Description of Additional Supplementary Files**

Supplementary Figures

**Supplementary Figure 1.** Characterization of dividing cells after targeted depletion of *Scgb1a1*^+^ cells.

**Supplementary Figure 2.** Characterization of mesenchymal cell activation after epithelial injury in SRC mice.

**Supplementary Figure 3.** Transcriptional changes in epithelial lung cells after *Scgb1a1*^+^ cell depletion.

**Supplementary Figure 4.** Characterization of DTA^+^ epithelial cells and comparison to transcriptional profiles of human lung diseases.

**Supplementary Figure 5.** Chemokine expression and cellular crosstalk in the SRC mouse model.

**Supplementary Figure 6.** Characterization of lung epithelial cells in the SfSRC mouse model.

**Supplementary Figure 7.** Trajectory analysis of lung epithelial cells in the SfSRC mouse model.

Supplementary DATA

**Supplementary Data 1: Detailed information for all scRNA-seq runs.**

Table shows cell type analyzed, mouse model, timepoint, number of mice analyzed, number of cells analyzed, filtering parameters, mean reads per cell, median genes per cell, and median UMI counts per cell.

**Supplementary Data 2: Marker genes for dividing cell populations in the lungs of SRC mice after two and three days of tamoxifen expression.**

Table shows AUC, power, average logFC, percentage of cells expressing the gene in the population (pct.1) and percentage of cells expressing the gene in the rest of the cells (pct.2). Markers were calculated using the roc test and pct.>0.2.

**Supplementary Data 3: Marker genes for mesenchymal cells in uninjured lungs (day 0) of SRC mice.**

Table shows AUC, power, average logFC, pct.1 and pct.2. Markers were calculated using the roc test and pct.>0.2.

**Supplementary Data 4: Marker genes for epithelial cells in uninjured lungs (day 0) of SRC mice.**

Table shows AUC, power, average logFC, pct.1 and pct.2. Markers were calculated using the roc test and pct.>0.2.

**Supplementary Data 5: Differentially expressed genes at day 2, 3, and 4 after tamoxifen administration in each epithelial population of SRC mice.**

Table shows p value, average logFC, pct.1, pct.2 and adjusted p value. DEGs were calculated using the MAST test, average logFC>2 and adjusted p value <0.05.

**Supplementary Data 6: Marker genes for epithelial cells on day 2 of SRC mice.**

Table shows AUC, power, average logFC, pct.1 and pct.2. Markers were calculated using the roc test and pct.>0.2.

**Supplementary Data 7: Common marker genes in DTA^+^ cells.**

Table shows common upregulated and downregulated genes of DTA+ epithelial cells as well as their Gene Set Enrichment Analysis. Common upregulated and downregulated genes were calculated by intersecting the DEGs (MAST test, min.pct=0.2, adjusted p value <0.05) of all DTA+ populations.

**Supplementary Data 8: Differentially expressed genes in DTA^+^-like cells of COVID-19 patients.**

Table shows p value, average logFC, pct.1, pct.2 and adjusted p value. DEGs were calculated using the MAST test, average logFC>1.5 and adjusted p value <0.05.

**Supplementary Data 9: Marker genes for epithelial cells in SfSRC mice (all timepoints merged).**

Table shows AUC, power, average logFC, pct.1 and pct.2. Markers were calculated using the roc test and pct.>0.2.

**Supplementary Data 10: DEGs during goblet to basal cell differentiation.**

Table shows q and Moran’s I values.

**Supplementary Data 11: DEGs during goblet to club cell differentiation.**

Table shows q and Moran’s I values.

**Supplementary Data 12: DEGs during club to ciliated cell differentiation.**

Table shows q and Moran’s I values.

**Supplementary Data 13: DEGs during club to AT2 cell differentiation.**

Table shows q and Moran’s I values.

**Supplementary Data 14: Ligand-receptor pairs used for crosstalk analysis.**

CellChat mouse database ligand-receptor pairs manually curated.
