# Supplementary material for: Single-cell division tracing and transcriptomics reveal cell types and differentiation paths in the regenerating lung
Source: Nat Commun. 2024 Mar 12;15:2246. doi: 10.1038/s41467-024-46469-4 (PMC10933319; doi:10.1038/s41467-024-46469-4)
Supplement: Supplementary file 18 — Reporting Summary [file 41467_2024_46469_MOESM18_ESM.pdf]

nature portfolio

Corresponding author(s): Leila R. Martins and Claudia Scholl

Last updated by author(s): Jan 20, 2024

Reporting Summary

Nature Portfolio wishes to improve the reproducibility of the work that we publish. This form provides structure for consistency and transparency in reporting. For further information on Nature Portfolio policies, see our Editorial Policies and the Editorial Policy Checklist.

Statistics

For all statistical analyses, confirm that the following items are present in the figure legend, table legend, main text, or Methods section.

n/a	Confirmed	
☐	☒	The exact sample size (*n*) for each experimental group/condition, given as a discrete number and unit of measurement
☐	☒	A statement on whether measurements were taken from distinct samples or whether the same sample was measured repeatedly
☐	☒	The statistical test(s) used AND whether they are one- or two-sided<br>*Only common tests should be described solely by name; describe more complex techniques in the Methods section.*
☒	☐	A description of all covariates tested
☐	☒	A description of any assumptions or corrections, such as tests of normality and adjustment for multiple comparisons
☐	☒	A full description of the statistical parameters including central tendency (e.g. means) or other basic estimates (e.g. regression coefficient) AND variation (e.g. standard deviation) or associated estimates of uncertainty (e.g. confidence intervals)
☐	☒	For null hypothesis testing, the test statistic (e.g. *F*, *t*, *r*) with confidence intervals, effect sizes, degrees of freedom and *P* value noted<br>*Give P values as exact values whenever suitable.*
☒	☐	For Bayesian analysis, information on the choice of priors and Markov chain Monte Carlo settings
☒	☐	For hierarchical and complex designs, identification of the appropriate level for tests and full reporting of outcomes
☐	☒	Estimates of effect sizes (e.g. Cohen's *d*, Pearson's *r*), indicating how they were calculated

Our web collection on statistics for biologists contains articles on many of the points above.

Software and code

Policy information about availability of computer code

Data collection	Microscopy images were acquired using NIS-Elements (Nikon) in a A1R (confocal fluorescence, Nikon) or Ni-E microscope (light microscopy, Nikon).<br>BD FACSDiva v8.0.x (BD Biosciences) was used during sorting.<br>NextSeq 500 (Illumina) (Fig. 1-3 and S1-3) or NovaSeq 6000 (Illumina) (all other figures) with the software version 1.7.0 were used for sequencing.
Data analysis	Microscopy images were analyzed using Fiji (ImageJ 1.53c).<br>FlowJo v10.7.1 (BD Biosciences) was used for flow cytometry analysis.<br><br>Processing of mass spectrometry data and statistical analysis:<br>Data analysis was carried out with MaxQuant v1.6.14.0 using an organism-specific database extracted from Uniprot.org under default settings. Identified false discovery rate (FDR) cutoffs were 0.01 on peptide level and on protein level. The match between runs (MBR) option was enabled to transfer peptide identifications across RAW files based on accurate retention time and m/z. The fractions were set in a way that MBR was only performed within each condition. Label-free quantification (LFQ) was done using a label free quantification approach based on the MaxLFQ algorithm. A minimum of two quantified peptides per protein was required for protein quantification. Adapted from the Perseus recommendations, protein groups with a non-zero LFQ intensity in 70% of the samples of at least one of the conditions were used for statistics. LFQ values were normalized via variance stabilization normalization. Based on the Perseus recommendations, missing LFQ values being completely absent in one condition were imputed with random values drawn from a downshifted (2.2 standard deviation) and narrowed (0.3 standard deviation) intensity distribution of the individual samples. For missing LFQ values with no complete absence in one condition, the R package missForest v1.4 was used for imputation.

Single-cell RNA sequencing analysis:
Processing of scRNA-seq data. Raw sequencing data was processed with 10x Genomics Cell Ranger v3.1.0. Reads were aligned to a custom reference genome, which was created based on the mouse mm10 reference genome v1.2.0 provided by 10x Genomics. Firstly, sequences for transgenes Sgcb1a1 (3’ of ER + 130 bp linker + 3’ UTR of Scgb1a1), Sftpc (rtTA-M2 coding sequence) and DTA were added to the reference genome FASTA file. Subsequently, the endogenous Esr1 (genomic position Chr10:4611989-5005614) and 3’ UTR of Scgb1a1 (genomic position Chr19:9083642-9083739) were masked from the genome. Then, three lines corresponding to the newly-added transgene sequences were added to the reference genome GTF file. Lastly, Cell Ranger was used to create the reference genome package from the modified FASTA and GTF files. SoupX v1.5.0 was used to remove ambient RNA contamination. To avoid underestimation of the global contamination fraction, manual gene lists were used for the sequencing runs of GFP+ mesenchymal cells on day 2 and 3 (Sftpc, Sftpa1, Sftpb, Sftpd, Dcn, Col1a1, Col1a2, Cldn3, Cldn18, Cldn2, Cldn4) and epithelial cells on day 4 (Sftpc, Sftpa1, Sftpb, Sftpd, Dcn, Scgb3a1, Foxj1) after tamoxifen administration in the SRC model. SoupX was otherwise run with default parameters. Further processing and analysis of count tables was performed with Seurat v3.2.1. Poor quality cells were filtered out based on high content of mitochondrial genes (4 to 7.5%, depending on the sample) and low total number of features (500 to 2500, depending on the sample), before integrating different samples. Detailed information for each scRNA-seq run can be found in Supplementary Data 1.

Sample integration, cell cycle regression, dimensionality reduction, clustering, and doublets exclusion. Individual samples were integrated with Seurat with IntegrateData() using 2,000 anchor features and integrating all common features between samples. Cell cycle phase was calculated by adapting the Seurat function CellCycleScoring() to use the GFP+ sorted cells as a reference. Cell cycle scores were then regressed out during data scaling with ScaleData() to mitigate the effects of cell cycle heterogeneity in the datasets from Fig. 3, 4, 7, Supplementary Fig. 3, 4, and 7. UMAP dimensionality reduction and nearest-neighbor graphs were calculated using the top 30 principle components. Cells were then clustered with FindClusters() with a resolution between 0.3 and 1.5, depending on cell number. Cell doublets were calculated with scDblFinder() (scDblFinder v1.2.0) using the default parameters and providing a vector of the runs id in the samples parameter. Clusters of cells composed of doublets were excluded from the analysis. Similar clusters were merged, taking into consideration the phylogenetic tree calculated with BuildClusterTree(). Cells were identified based on the expression of known marker genes and, when present, clusters composed of endothelial (Pecam+) or hematopoietic (Ptprc+) cells were excluded from the analysis. In the case of mesenchymal cell analysis, only clusters expressing Col1a1 were kept.

Differential gene expression and gene set enrichment analysis. Differential gene expression between different cell types (markers) was calculated with FindMarkers(test.use=“roc”, only.pos=TRUE, min.pct=0.2), and differentially gene expression between cells from non-injured and injured lungs was calculated with FindMarkers(test.use="MAST", min.pct=0.2). Common marker genes of DTA+ epithelial cells were calculated separately for downregulated and upregulated genes by intersecting the DEGs previously calculated for each population with FindMarkers(test.use="MAST”, ident.1=”DTA+_cell_type”, ident.2=c(“all populations excluding DTA+ cells”, min.pct=0.2)). Only DEGs with a p_adj <0.05 were considered. Heatmaps for DEGs were generated with Seurat’s function DoHeatmap(). GSEA was performed using Metascape v3.5 with a p-value cutoff of 10E-6. Activities of 14 pathways were inferred with PROGENy v1.10.0 using organism=”Mouse”, scale=FALSE and otherwise default values. Scores were scaled and centered using Seurat’s ScaleData(). Heatmap showing pathway activity for each cell cluster at day 2 was drawn with ComplexHeatmap v2.4.3.

Transcriptome correlation between cell types. For every cell type combination of day 2 samples, Spearman's rank correlation coefficient was calculated from the average normalized expression of all genes. Heatmap was drawn with ComplexHeatmap v2.4.3.

Expression of human lung diseases signatures. To infer the expression of human lung diseases signatures in mouse epithelial cells, SRC mouse features were converted to their human orthologs using bioDBnet and a humanized Seurat object was generated using the same parameters as for the mouse object. Single-cell scores were calculated with AddModuleScore() using gene signatures for asthma, non-small cell lung carcinoma, and influenza from DisGeNET v7, and chronic obstructive pulmonary disease, COVID-19 bronchial epithelial cells, and pulmonary fibrosis from MSigDB v7.5.1 databases. Expression of the different signatures by each SRC epithelial population at day 0 and day 2 was drawn with the Seurat’s RidgePlot() function.

Integration with COVID-19 dataset and DEGs analysis. Humanized Seurat objects from SRC mouse epithelial cells at day 0 and day 2 were generated by replacing mouse genes with their human orthologs. Conversion from mouse to human genes was done using bioDBnet. Epithelial cells from controls and COVID-19 patients, selected based on the author’s “Epithelial cells” annotation were used to integrate with our mouse datasets following Seurat package guidelines. Seurat objects for each patient sample and each mouse run were normalized and FindVariableFeatures() was run before calculating anchors. To identify integration anchors, FindIntegrationAnchors() was run with a k.filter of 100 (due to the low cell number in some patient samples) and SRC mouse samples were used as reference. Integration anchors were then used to integrate the objects with IntegrateData(). The integrated Seurat object was scaled, UMAP dimensional reduction and nearest-neighbor graphs were calculated using the top 30 principle components, and clusters were calculated with FindClusters() and a resolution of 0.5. Cell type identification of the COVID-19 dataset was done according to the authors’ annotation (“cell_type_fine” identity class) and clusters were annotated based on the identity of the majority of the cells in the cluster. When several clusters with the same cell type were identified, a number was added as a suffix. The cluster containing mouse DTA+ cells, together with DTA+-like cells from the COVID-19 dataset, was annotated as DTA+-like cells. DEGs in DTA+-like cells were calculated with FindMarkers(test.use=”MAST, min.pct=0.2, ident.1=”DTA+-like cells”) and considering only epithelial cells from COVID-19 patients (excluding cells from the SRC dataset and from human controls). Only DEGs with a p_adj <0.05 were considered.

Similarity to previously described epithelial cell types. Marker genes for all SfSRC mouse epithelial cell populations using all timepoints were calculated with FindMarkers(test.use="MAST", logfc.threshold=-Inf, min.pct=-Inf), and housekeeping genes listed in Laughney et al were excluded. Genes were ranked according to average log FC, and the top 50 genes from previously described epithelial cell types were used to calculate the normalized enrichment score (NES) for each SfSRC mouse epithelial population using fgsea v1.14.0 package.

Projection of cells between mouse models and experiments. To compare the cell populations present in different experiments, SingleCellExperiment objects were generated and cells from one experiment were projected onto cells from another experiment using scmapCell(). Cell assignment was done with scmapCell2Cluster() and drawn with getSankey() from scmap v1.10.0 package.

Expression of chemokines. Chemokine genes considered for analysis were taken from MGI GO TERM “Chemokine activity”. scRNA-seq data to assess chemokine expression in homeostasis by lung cell types, including mesenchymal, immune, and endothelial cells, was taken from the

Mouse Cell Atlas (Supplementary Fig. 5c).

Cell-cell communication analysis. Intercellular interactions were inferred in mesenchymal (Fig. 2 cells) and epithelial cells (Fig. 4 cells) of the SRC model before (day 0) and after tamoxifen administration (day 2) with CellChat v1.1.0 following the official workflow and using standard parameters. The analysis was based on expression of ligand-receptor pairs from the CellChat mouse database, which we manually adjusted to exclude ligand-receptor pairs not supported by literature and to include interactions playing a role in intercellular junctions and mesenchymal cells of the lung (adjusted database is provided in Supplementary Data 14). Neuroendocrine cells were excluded from the analysis due to low cell numbers. For each timepoint, preprocessing of normalized count tables was performed with identifyOverExpressedGenes(), identifyOverExpressedInteractions() and projectData(). The cell-cell communication network was inferred using computeCommunProb(), which by default requires 25% of the cells per group to express the ligand or receptor gene. Summarizing analyses were performed with computeCommunProbPathway(), aggregateNet() and netAnalysis_computeCentrality(). The summed incoming and outgoing interactions strengths were obtained with netAnalysis_signalingRole_scatter() and scaled to the maximum summed interaction strength at the respective time point. For comparison of the signaling pathways between the two timepoints, the CellChat objects were merged with liftCellChat() and mergeCellChat().

Force-directed graph and PAGA analysis. Connectivities between different cell types were analyzed with the Scanpy package. PCA and a neighborhood graph were computed with pp.pca() and sc.pp.neighbors() using n_neighbors=10 and n_pcs=20. The force-directed graph was drawn with sc.tl.draw_graph(). For the PAGA analysis, the data was restricted to non-dividing cell clusters, on which the PCA and neighborhood graph were re-calculated using the same functions and parameters as above. Connectivities were quantified with tl.paga().

Diffusion maps. Diffusion maps restricted to cell type subsets of interest were calculated with Scanpy. The data was restricted to the cell type subset and the top 2,000 variable genes with a minimum count of 10. To avoid obtaining cell cycle-related genes, dividing cell clusters and cells in the G2/M or S phase were removed for the calculation of the top variable genes. PCA and the neighborhood graph were calculated with pp.pca() and sc.pp.neighbors() using n_neighbors=15 and n_pcs=20. The diffusion map was calculated with tl.diffmap().

RNA velocity. Spliced and unspliced read counts per gene were obtained with Velocyto v0.17.17 and merged with the pre-processed normalized and log-transformed count data from Seurat. To reduce the influence of possible variable kinetic rates between different cell types or states, RNA velocities were calculated separately on a reduced data set containing only cell types along the transition pathway using scVelo v0.2.2. First and second order moments were calculated using scvelo.pp.moments() with default parameters, velocities were calculated with scvelo.tl.velocity() in "dynamical" mode, and a velocity graph was constructed with scvelo.tl.velocity_graph(). The velocities were projected and visualized on diffusion map embeddings (see above) using scvelo.pl.velocity_embedding_grid(density = 0.5).

Trajectory inference and differential expression analysis. In the diffusion maps, trajectory analysis was performed with Monocle 3 v0.2.2 using default parameters, unless otherwise specified. The scale of the diffusion map DC values was adjusted by multiplying by 100. Cells in the diffusion map were clustered using cluster_cells() with parameters partition_qval=0.05 and num_iter=1. Trajectories were inferred using learn_graph(). Cells were assigned a pseudotime with the order_cells() function, for which root cells were manually chosen according to prior knowledge. The differential expression analysis was manually restricted to cells along the respective branch of interest and carried out using the function graph_test() with the parameter neighbor_graph="principal_graph". The expression was then scaled to the cell subset and smoothed using the loess() function in R with span set to 0.5. Heatmaps of the top 1,000 DEGs (sorted by q-value and Morans's I) were drawn with ComplexHeatmap v2.4.3. Mouse transcription factors in the DEGs were annotated with AnimalTFDBv3.0.

For manuscripts utilizing custom algorithms or software that are central to the research but not yet described in published literature, software must be made available to editors and reviewers. We strongly encourage code deposition in a community repository (e.g. GitHub). See the Nature Portfolio guidelines for submitting code & software for further information.

Data

Policy information about availability of data

All manuscripts must include a data availability statement. This statement should provide the following information, where applicable:

- Accession codes, unique identifiers, or web links for publicly available datasets
- A description of any restrictions on data availability
- For clinical datasets or third party data, please ensure that the statement adheres to our policy

Gene signatures for asthma, non-small cell lung carcinoma, and influenza were obtained from DisGeNET v7, and gene signatures for chronic obstructive pulmonary disease, COVID-19 bronchial epithelial cells, and pulmonary fibrosis from MSigDB v7.5.1 databases. Processed single-nuclei RNA-seq data from lungs of COVID-19 and control patients was obtained through the Gene Expression Omnibus (GEO) database under the accession code GSE171524. A manually curated database of ligand-receptor pairs was generated from the CellChat mouse database, and is provided in Supplementary Data 14. Chemokine genes considered for analysis were taken from MGI GO TERM "chemokine activity". scRNA-seq reads were aligned to a custom reference genome published with the DOI 10.5281/zenodo.10478745. Proteome ID UP000000589 from Uniprot was used for mass-spectrometry data analysis. The mass spectrometry proteomics data generated in this study have been deposited in the ProteomeXchange Consortium via the PRIDE partner repository with the dataset identifier PXD039508. The scRNA-seq data generated in this study have been deposited in the GEO database with under the accession code GSE223816.

Research involving human participants, their data, or biological material

Policy information about studies with human participants or human data. See also policy information about sex, gender (identity/presentation), and sexual orientation and race, ethnicity and racism.

Reporting on sex and gender	N/A
Reporting on race, ethnicity, or other socially relevant groupings	N/A

Population characteristics	N/A
Recruitment	N/A
Ethics oversight	N/A

Note that full information on the approval of the study protocol must also be provided in the manuscript.

Field-specific reporting

Please select the one below that is the best fit for your research. If you are not sure, read the appropriate sections before making your selection.

- [x] Life sciences
- [ ] Behavioural & social sciences
- [ ] Ecological, evolutionary & environmental sciences

For a reference copy of the document with all sections, see nature.com/documents/nr-reporting-summary-flat.pdf

Life sciences study design

All studies must disclose on these points even when the disclosure is negative.

Sample size	No statistical method was used to predetermine sample size.<br>Sample size for scRNA-seq experiments was determined considering the severity of the lung injury and the percentage of GFP+ cells, so enough cells could be sorted for sequencing. All immunofluorescence analyses were done in 3 mice per timepoint. Masson staining was done in 3 mice per timepoint. H&E staining of SfSRC lungs was done in 2 mice per timepoint.<br>Sample size was considered sufficient based on the high reproducibility of the results.
Data exclusions	No data were excluded.
Replication	Immunofluorescence analyses where performed in 5 independent experiments in the SRC mouse line, 3 independent experiments in the SfSRC mouse line, and in 1 experiment in the RC control line. In all experiments, 3 animals were analyzed per group.<br>Masson staining was performed in 2 independent experiments. H&E staining of SfSRC lungs was done once.<br>Mass spectrometry analysis was done using 3 independent experiments. Lung organoid cultures were performed twice.<br>Immunofluorescence, histology, mass spectrometry, and lung organoid culture results were reproducible in all experiments done.<br>scRNA-seq findings, such as the presence of DTA+ cells, and type of dividing cells were consistent between experiments.
Randomization	Mice were randomly distributed across experimental groups.
Blinding	Investigators were not blinded since knowledge of experimental groups was essential for single cell sample preparation and analyses.

Reporting for specific materials, systems and methods

We require information from authors about some types of materials, experimental systems and methods used in many studies. Here, indicate whether each material, system or method listed is relevant to your study. If you are not sure if a list item applies to your research, read the appropriate section before selecting a response.

Materials & experimental systems

n/a	Involved in the study
[ ]	[x] Antibodies
[x]	[ ] Eukaryotic cell lines
[x]	[ ] Palaeontology and archaeology
[ ]	[x] Animals and other organisms
[x]	[ ] Clinical data
[x]	[ ] Dual use research of concern
[x]	[ ] Plants

Methods

n/a	Involved in the study
[x]	[ ] ChIP-seq
[ ]	[x] Flow cytometry
[x]	[ ] MRI-based neuroimaging

Antibodies

Antibodies used	Immunofluorescence:<br>Acetylated tubulin (Sigma, T7451, clone 6-11B-1, lot# 059M4812V), Agr2 (Proteintech, 12275-1-AP, lot# 58183), CC10 (Millipore, 07-623, lot# 3145007), CD34 (BD Biosciences, 553731, clone RAM34, lot# 7075697), Cyp2f2-Alexa Fluor 647 (Santa Cruz, sc-374540 AF647, clone F-9, lot# D1218), GFP-FITC (Rockland, 600-102-215, lot# 31007),<br>Krt5 (Abcam, ab53121, lot# GR38774-37), Lamp3 (Dendritics, DDX0192-100, clone 1006F7.05, lot# DDX0192-030), Npnt (Invitrogen, PA547610, lot# VE2996317), Pdpn (Abcam, ab11936, clone RTD4E10, lot# GR3273681-3), SPC (Millipore, #AB3786, lot# 3537117),<br>donkey anti-rabbit Alexa Fluor 568 (Invitrogen, A10042, lot# 1757124), goat anti-rat Alexa Fluor 568 (Invitrogen, A11077, lot# 1753597), and goat anti-hamster Alexa Fluor 647 (Invitrogen, A21451, lot# 1631002).

	Flow cytometry:<br>CD31 APC (BD Biosciences, 551262, clone MEC 13.3, lot# 8174772), CD45 APC (BD Biosciences, 559864, clone 30-F11, lot# 7166674), Ter119 APC (eBioscience, 17-5921-82, clone TER-119, lot# 4348147), Epcam PE-Cy7 (BD Biosciences, 118216, clone G8.8, lot# B309548), CD24 PE (eBioscience, 12-0242-81, clone M1/69, lot# E01148-1635), MHCII Alexa Fluor 700 (eBioscience, 56-531-82, clone M5/114.15.2, lot# 1919519), Npnt (Invitrogen, PA547610, lot# VE2996317), Pdgfra BV711 (BD Biosciences, 740740, APA5), Sca-1 APC-Cy7 (BD Biosciences, 560654, clone D7, lot# 8304556), CD34 PerCP-Cy5.5 (Biolegend, 119327, clone MEC14.7, lot# B286852), donkey anti-goat Alexa Fluor 680 (Invitrogen, A32860, lot# UB282073).
Validation	Immunofluorescence:<br>Acetylated tubulin (Sigma, T7451), Agr2 (Proteintech, 12275-1-AP), CC10 (Millipore, 07-623), SPC (Millipore, #AB3786),CD34 (BD Biosciences, 553731), Cyp2f2-Alexa Fluor 647 (Santa Cruz, sc-374540 AF647), GFP-FITC (Rockland, 600-102-215), Krt5 (Abcam, ab53121), Lamp3 (Dendritics, DDX0192-100), Npnt (Invitrogen, PA547610), and Pdpn (Abcam, ab11936) were validated by the manufacturer and used in previous studies (see data sheets).<br><br>Flow cytometry:<br>CD31 APC (BD Biosciences, 551262), CD45 APC (BD Biosciences, 559864), Ter119 APC (eBiosciences, 17-5921-82), CD24 PE (eBiosciences, 12-0242-81), CD34 PerCP-Cy5.5 (Biolegend, 119327), MHCII Alexa Fluor 700 (eBiosciences, 56-531-82), Epcam PECy7 (BD Biosciences, 118216), Sca-1 APC-Cy7 (BD Biosciences, 560654), and Pdgfra BV711 (BD Biosciences, 740740) were validated for flow cytometry by the manufacturer and used in previous studies (see data sheets).

Animals and other research organisms

Policy information about studies involving animals; ARRIVE guidelines recommended for reporting animal research, and Sex and Gender in Research

Laboratory animals	Mice were housed under strict specified pathogen-free (SPF) conditions at 22.0 ± 2.0 °C and 55.0 ± 10.0 % relative humidity. The light/dark cycle was adjusted to 14 hours lights on and 10 hours lights off with the beginning of the light and dark period set at 6 am and 8 pm, respectively. Mice of both sexes between 8 and 16 weeks-old were used. Scgb1a1tm1(cre/ERT)Blh/J (Scgb1a1-CreER; C57BL/6N background), B6.129P2-Gt(ROSA)26Sortm1(DTA)Lky/J (Rosa26R-DTA; C57BL/6 background), and Tg(Pgk1-Ccnb1/EGFP)1Aklo/J (CycB1-GFP; C57BL/6J background) mice were purchased from The Jackson Laboratory and used to generate the SRC mouse line. Sftpctm1(cre/ERT2,rtTA)Hap (Sftpc-CreER) mice, kindly provided by Rocio Sotillo, DKFZ, Heidelberg, Germany (originally provided by Harold A. Chapman, University of California, San Francisco, USA), were crossed with SRC mice to generate the SfSRC mouse line. Rosa26R-DTA x CycB1-GFP (RC) mice were used as controls. The mice were homozygous for all transgenes except CycB1-GFP, which has multiple transgenes integrated in the genome.
Wild animals	The study did not involve wild animals.
Reporting on sex	Both male and female mice were used.
Field-collected samples	The study did not involve samples collected from the field.
Ethics oversight	All animal procedures were approved by the regional authority in Karlsruhe, Germany (reference numbers 35-9185.81/G-238/14 and 35-9185.81/G-303/19) and performed according to federal and institutional guidelines.

Note that full information on the approval of the study protocol must also be provided in the manuscript.

Plants

Seed stocks	N/A
Novel plant genotypes	N/A
Authentication	N/A

Flow Cytometry

Plots

Confirm that:

- [x] The axis labels state the marker and fluorochrome used (e.g. CD4-FITC).
- [x] The axis scales are clearly visible. Include numbers along axes only for bottom left plot of group (a 'group' is an analysis of identical markers).
- [x] All plots are contour plots with outliers or pseudocolor plots.
- [x] A numerical value for number of cells or percentage (with statistics) is provided.

Methodology

Sample preparation	PBS-perfused lungs were inflated with 2 ml of digestion cocktail containing 50 U/ml dispase (Corning), 250 U/ml collagenase type I (Worthington), 5 U/ml elastase (Worthington), and 60 U/ml DNAse I (Roche). Trachea was clipped distally and lungs were dissected in a petri dish on ice to remove extrapulmonary airways (trachea and main bronchi). Lung lobes were placed in a C tube (Miltenyi) containing 3 ml of digestion cocktail, and the m_lung_01 program was run on gentleMACS (Miltenyi). C tubes were placed in a rotating incubation oven at 37ºC for 30 minutes. The m_lung_02 program was run again, and the tubes were placed on ice for the next steps. The lung cells were passed through a 70 µm cell strainer (Corning) and centrifuged at 400 g for 5 minutes. The pellet was resuspended in a red blood lysis buffer solution (0.15 M NH_4Cl, 10 mM $KHCO_3$, 0.1 mM EDTA), incubated for 2 minutes on ice, and washed with EasySep buffer (STEMCELL Technologies) at 400 g for 5 minutes. To isolate mesenchymal cells (Fig. 2, Supplementary Fig. 2), a milder digestion cocktail was used containing only 375 U/ml collagenase and 60 U/ml DNase I. Lung single-cell suspensions were resuspended in EasySep buffer, incubated with primary antibodies for 20 minutes at 4ºC, washed with EasySep buffer at 400 g for 5 minutes, and, in the case of unconjugated primary antibodies, secondary antibodies were added, incubated for 15 minutes at 4ºC and washed again.
Instrument	Cell sorting was performed with FACSAria II (BD Bioscience).
Software	BD FACSDiva v8.0.x (BD Biosciences) was used during sorting, and FlowJo v10.7.1 (BD Biosciences) was used for posterior analysis.
Cell population abundance	After excluding endothelial and hematopoietic cells, GFP+ cells represented 0.034% to 0.16% of total cells (Supplementary Fig. 1a). Epithelial cells (EPCAM+) had 0.043% to 3.72% of GFP+ cells (Supplementary Fig. 3a and 6c).
Gating strategy	Single-cell RNA sequencing:<br>Debris were excluded by FSC-A vs SSC-A, and singlets selected based on FSC-A vs FSC-W or FSC-A vs FSC-H profiles. Dead (DAPI+), hematopoietic (CD45+ and Ter119+) and endothelial (CD31+) cells were excluded. Dividing cells were sorted based on their expression of GFP (Supplementary Fig. 1a). For epithelial cell analysis, EPCAM+ cells and dividing EPCAM+ GFP+ cells were sorted. To enable enrichment of infrequent cell types, AT2 cells were partially depleted using an anti-MHC II antibody (Supplementary Fig. 3a), or AT2 and ciliated cells were partially depleted using an anti-CD24 antibody (Supplementary Fig. 4a). GFP+ AT2 or ciliated cells were not depleted as they were isolated during the separate GFP+ cell sorting.<br><br>Lung organoids:<br>Progenitor epithelial cells (DAPI- CD45- CD31- Ter119- EPCAM high CD24 dim) were cultured with adventitial fibroblasts (DAPI- CD45- CD31- Ter119- EPCAM- PDGFRA+ CD34+ SCA-1+) or alveolar fibroblasts (DAPI- CD45- CD31- Ter119- EPCAM- CD34- SCA-1- PDGFRA+ NPNT+) (Supplementary Fig. 2c).<br><br>Mass spectrometry:<br>Adventitial fibroblasts (DAPI- CD45- CD31- Ter119- EPCAM PDGFRA+ CD34+ Sca-1+), alveolar fibroblasts (DAPI- CD45- CD31- Ter119- EPCAM- CD34- Sca-1- PDGFRA+), and PDFGRA- cells (DAPI- CD45- CD31- Ter119- EPCAM- CD34- Sca-1- PDGFRA-) were sorted from uninjured lungs.

- [x] Tick this box to confirm that a figure exemplifying the gating strategy is provided in the Supplementary Information.
